# Effect of Different Loads on Stroke and Coordination Parameters During Freestyle Semi-Tethered Swimming

**DOI:** 10.2478/v10078-012-0021-9

**Published:** 2012-05-30

**Authors:** Rocio Dominguez-Castells, Raul Arellano

**Affiliations:** 1 Department of Physical Education and Sport, Faculty of Sport Sciences, University of Granada, Spain.

**Keywords:** intra-cycle speed, propulsive phases, index of coordination, resisted training

## Abstract

The aim of this study was to analyse to what extent the use of different loads modifies freestyle stroke and coordination parameters during semi-tethered swimming, and to examine whether those changes are positive or negative to swimming performance. First, behaviour of swimming speed (v), stroke rate (SR) and stroke length (SL) with increasing loads was examined. Secondly, mean and peak speed of propulsive phases (propv_mean_ and propv_peak_) were analysed, as well as the relative difference between them (%v). Finally, index of coordination (IdC) was assessed. Eighteen male swimmers (22.10±4.31years, 1.79±0.07m, 76.74±9.00kg) performed 12.5m maximal sprints, pulling a different load each trial (0, 1.59, 2.21, 2.84, 3.46, 4.09, 4.71, 5.34, 5.96, 6.59, 7.21 and 7.84kg). Rest between repetitions was five minutes. Their feet were tied together, keeping a pull-buoy between legs and isolating the upper limb action. A speedometer was used to measure intra-cycle speed and the test was recorded by a frontal and a lateral underwater cameras. Variables v and SL decreased significantly when load increased, while SR remained constant (p<0.05). Propv_mean_ and propv_peak_ decreased significantly with increasing loads (p<0.05). In contrast, %v grew when load rose (r = 0.922, p<0.01), being significantly different from free swimming above 4.71kg. For higher loads, swimmers did not manage to keep a constant velocity during a complete trial. IdC was found to increase with loads, significantly from 2.84kg (p<0.05). It was concluded that semi-tethered swimming is one training method useful to enhance swimmers’ performance, but load needs to be individually determined and carefully controlled.

## Introduction

In swimming, race time can be divided into four components: start time, swimming time, turn time and finish time (Arellano et al., 1994). Regarding actual swimming, the time needed to complete one lap can be considered as a function of stroke rate and stroke length. As in other cyclical activities, swimmers need to find the optimal compromise between stroke rate and stroke length to attain and keep the maximal velocity during a race (Alberty et al., 2005).

Numerous studies have been carried out to observe and understand the evolution of this “SL × SR” model during competitive events (Arellano et al., 1994; Chollet et al., 1997; Craig et al., 1985). Throughout the race, as fatigue develops, speed and stroke length decrease whereas stroke rate remains constant or slightly increases at the end of the race (Alberty et al., 2009; Chollet et al., 1997; Craig et al., 1985; Hay, 2002; Keskinen and Komi, 1993). Swimmers can choose different strategies to develop their maximal speed as a function of the race distance and they attempt to maintain this chosen speed in spite of fatigue throughout the race.

Stroke rate and stroke length combinations (and, therefore, speed values) are determined by several factors such as anthropomorphic variables, muscle strength, physical conditioning and swimming economy (Pelayo et al., 2007). Another factor with big influence on swimming speed is load (Shionoya et al., 1999). In the latter study, they assessed speeds from 1.34m/s with 1kg load to 0.45m/s with 10kg load, but stroking parameters were not studied. To our knowledge, only one recent study has analysed speed, stroke rate and stroke length while semi-tethered swimming with increasing resistances (Gourgoulis et al., 2010).

In contrast, swimming speed during propulsive stroke phases has not been previously studied under resisted conditions. Considering the stroke phases proposed by Chollet et al., (2000), we can distinguish two propulsive phases (pull and push) and two non-propulsive ones (entry-catch and recovery). Regardless of every individual combination of stroke rate and stroke length, swimming speed is expected to be higher during propulsive phases in both free and semi-tethered swimming. Intra-cycle velocity variations were studied at different swimming paces (Schnitzler et al., 2010) and while swimming with parachute (Schnitzler et al., 2011), but not with different loads. To the authors’ knowledge, only one study (Telles et al., 2011) has examined changes in index of coordination (IdC) in three different resisted swimming conditions.

Therefore, the aim of the present study was to analyse to what extent the use of different loads modifies freestyle stroke and coordination parameters during semi-tethered swimming, and to examine whether those changes are positive or negative to swimming performance. With this analysis it was intended to bring light to the value of semi-tethered swimming for training purposes.

## Materials and Methods

### Participants

A group of 18 male college swimmers volunteered to participate in our study (mean age 22.10±4.31years, stature 1.79±0.07m, arm span 1.85±0.08m and body mass 76.74±9.00kg). All of them had trained in swimming for at least 5 years and had competed at regional or national level (25m time, in-water start =14.84±1.21s). The protocol was fully explained to them before they provided written consent to participate in the study, which was approved by the university ethics committee.

### Procedures

The test was conducted in one swimming pool session, at the end of the competitive season. It consisted in 12.5m swimming across the pool, at maximal speed, pulling a different load each trial, which was added by means of a pulley system. The swimmers rested five minutes between two consecutive repetitions. After a standardized 800m warm-up, first load was 4.5kg and it increased 2.5kg each trial. Considering the pulley system effects (mechanical advantage, friction and components weight), real loads pulled by the swimmers were 0, 1.59, 2.21, 2.84, 3.46, 4.09, 4.71, 5.34, 5.96, 6.59, 7.21 and 7.84kg. This was checked prior to the test, in the same conditions. Swimmers were connected to the load by means of a rope and a belt. Their feet were tied together, keeping a pull-buoy between legs and isolating the upper limb action. They were asked not to breathe during each trial to keep head position constant.

### Measurements

A speedometer attached to the swimmer’s belt was used to measure intra-cycle swimming speed (Sportmetrics S.L., Spain, frequency: 200 Hz, accuracy: 0.1mm). The test was recorded by a frontal and a lateral underwater cameras (Sony, frequency: 50 Hz, shutter speed: 1/250s), fixed to the pool wall.

### Analysis

Intra-cycle speed was recorded for every participant and trial. It was sampled at a frequency of 200 Hz and subsequently smoothed with a low-pass Butterworth filter with a cut-off frequency of 5 Hz. For each trial, three middle strokes were selected to avoid both the effect of the impulse from the wall and the speed decrease at the end. One stroke started when one hand first touched the water while entering it and finished the next time the same event happened for the same hand. Mean speed (v) was calculated for these 3 strokes. Stroke rate (SR) was calculated from the 3 strokes time:

SR (Hz)=number of strokes/strokes time (s)

Then, stroke length (SL) was obtained with the following equation:

$$\mathrm{SL}(m/\mathrm{cic})=\frac{v(m/s)}{\mathrm{SR}(\mathrm{Hz})}$$

Average of every variable for the whole group and every single load was calculated and represented. Intra-cycle speed curves were compared among swimmers and loads, to try to find any repeated patterns.

Within the stroke phases defined by Chollet et al. (2000), ‘pull’ and ‘push’ were considered the propulsive ones. ‘Pull’ phase starts after the hand′s entry into the water, when it reaches the most forward point and begins to move backwards. It ends when the hand is under the shoulder, on an imaginary vertical line. Here begins the ‘push’ phase, which ends at the moment the hand is completely out of water. With intra-cycle speed and video images mean and peak speed for the propulsive phases (pull and push) in three strokes (propv_mean_ and propv_peak_, respectively) were obtained for each trial and swimmer. In addition, percentage of increase from propv_mean_ to propv_peak_ (%v) was calculated. This variable was used as an indicator of propulsive intra-cycle velocity fluctuations magnitude. Video analysis allowed us to calculate index of coordination (IdC) for every trial. As for the stroke parameters, average IdC, propv_mean_, propv_peak_ and %v for the group and every load were calculated and represented.

### Statistical analysis

Descriptive statistics was used to calculate means and standard deviations. All variables (v, SR, SL, propv_mean_, propv_peak_, %v and IdC) were tested for normality (Shapiro-Wilk test). After performing Levene’s test for variance homogeneity, one-way repeated measures ANOVA was used to assess differences among loads for every variable. A two-way ANOVA was used to compare propv_mean_ and propv_peak_ along the test. Finally, Pearson’s correlation coefficients were calculated between load and the rest of variables. The statistical analysis was carried out using a statistical software package (SPSS 15.0). Statistical significance was set at p<0.05.

## Results

**Figure 1:**
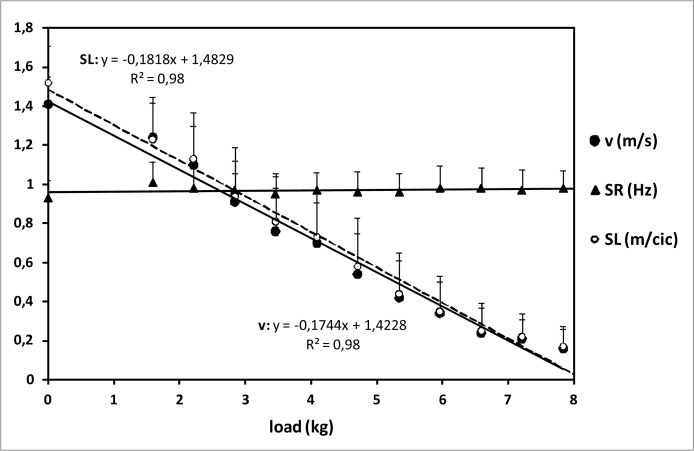
Behavior of some stroking parameters during semi-tethered swimming. Error bars are standard deviation (SD).

Behavior of v, SR and SL during semi-tethered swimming with increasing loads is represented in Figure 1. Stroke rate did not change significantly when load did (0.97±0.02Hz). In contrast, v and SL decreased with increasing loads (r = −0.985, −0.989, respectively, p<0.01) (Table 1). Range of values was: v: 1.41–0.16m/s; SL: 1.52–0.17m/cic.

**Figure 2:**
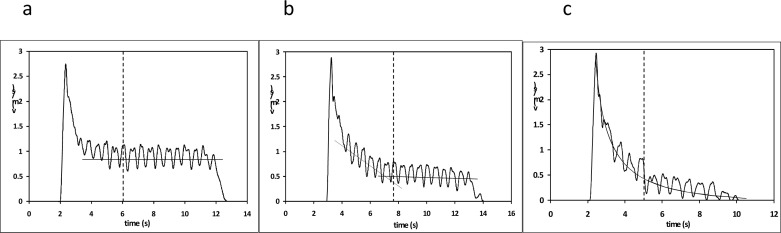
Behavior patterns of intra-cycle speed while semi-tethered swimming. a) 4.09kg load; b) 5.96kg load; c) 7.84kg load. The analysis started from the dotted line.

When comparing intra-cycle speed curves among participants and loads three main patterns were observed (Figure 2). Regardless of the impulse from the wall, speed followed a horizontal trend for the first six loads (until 4.71kg) (Fig. 2a). For the next two loads (5.34–5.96kg) speed decreased progressively in the first part of the trial and then remained constant in the second part (Fig. 2b). Finally, for the highest loads (6.59kg and higher) speed described a concave upward curve, dropping quickly at the beginning and more gradually at the end, until reaching 0m/s (Fig. 2c).

**Figure 3:**
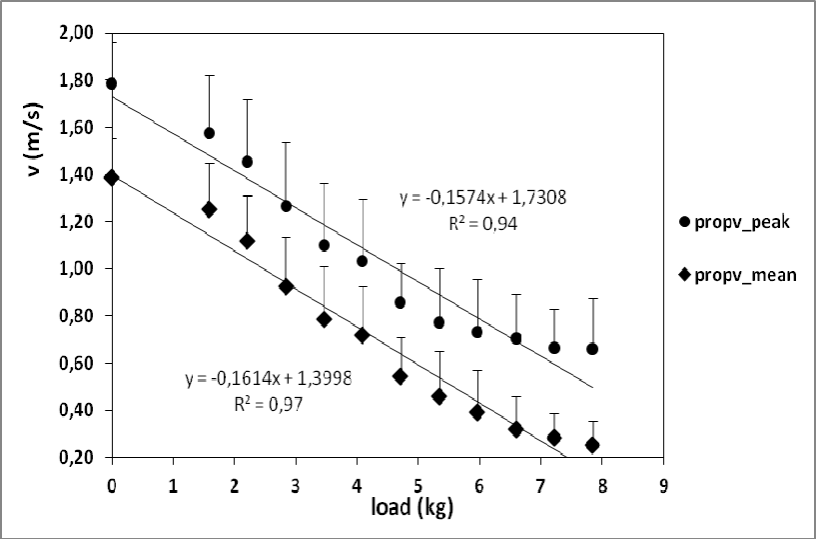
Mean and peak speed of propulsive phases (pull+push) while semi-tethered swimming. Error bars are standard deviation (SD).

Variable propv_peak_ was significantly higher than propv_mean_ (p<0.05) and they were positively correlated (r = 0.995, p<0.01). Mean speed in propulsive stroke phases (propv_mean_) decreased significantly with increasing loads in semi-tethered swimming (r = −0.984, p<0.01) (Table 1), from 1.39±0.17m/s with 0kg to 0.25±0.10m/s with 7.84kg load (Figure 3). Peak speed (propv_peak_) dropped significantly from 1.79±0.17m/s with 0kg to 0.73±0.22m/s with 5.96kg load (first nine loads) and did not change significantly for the highest loads (r = −0.971, p<0.01).

**Figure 4:**
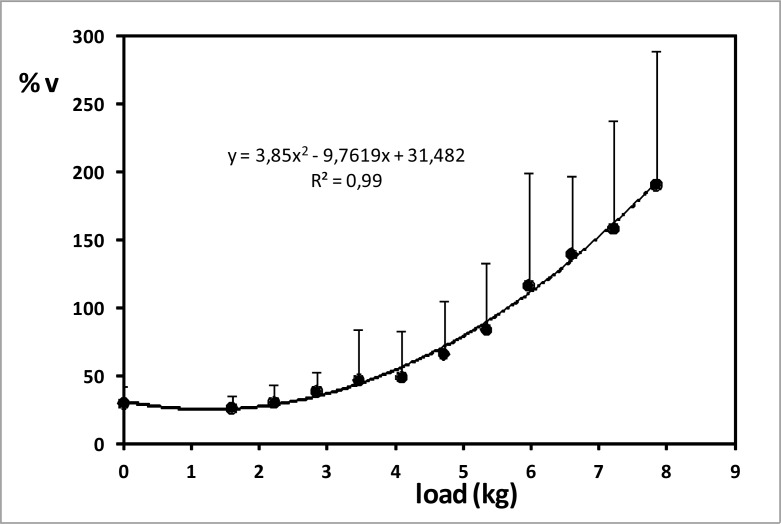
Percentage of increase from mean to peak propulsive speed during semi-tethered swimming. Error bars are standard deviation (SD).

Percentage of increase from mean to peak speed in the propulsive phases (%v) did not undergo any significant changes neither from 0kg to 4.09kg load (first six trials; %v = 36.94±9.57%) nor from 6.59kg to 7.21kg load (%v = 149.23±13.21%) (Figure 4). In contrast, it increased significantly and in a quadratic way when load raised between 4.09kg and 6.59kg and from 7.21kg to 7.84kg, when it almost reached 200% (r = 0.922, p<0.01). Consistently, propv_mean_ and propv_peak_ were negatively correlated with %v (r = −0.871, −0.824, respectively, p<0.01).

**Figure 5:**
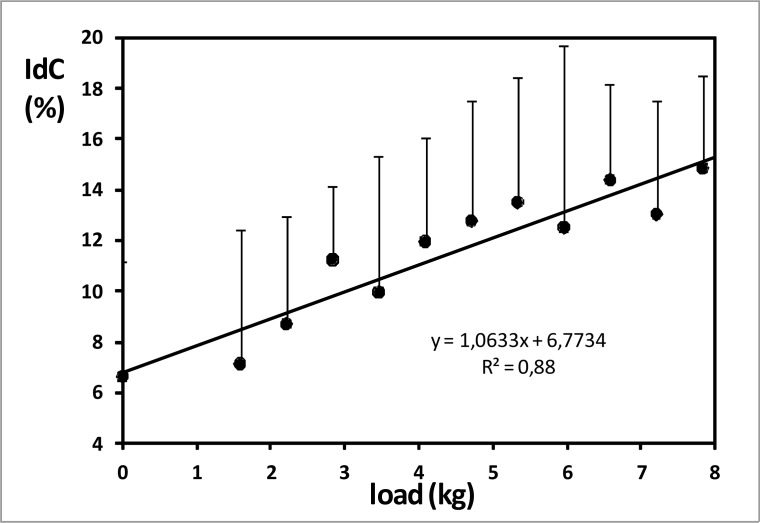
Index of coordination during semi-tethered swimming. Error bars are standard deviation (SD).

Coordination mode used in free and semi-tethered swimming was superposition (IdC>0%). IdC was 6.6±4.6% when swimming free and it increased significantly with loads (p<0.05), from 7.1±5.3% with 1.59kg to 14.8±3.7% with 7.84kg (Figure 5). High positive significant correlation was found between load and IdC (r = 0.910, p<0.01).

## Discussion

The aim of the present study was to analyse the effect of different loads on freestyle stroke and coordination parameters during semi-tethered swimming and to examine whether those changes are positive or negative to swimming performance. The main findings of our study showed that percentage of increase from mean to peak speed in the propulsive phases grew following a quadratic trend with increasing loads. Besides, IdC rose significantly with load. Three different intra-cycle velocity patterns were noticed throughout loads.

Swaine and Reilly (1983) stated that freely chosen stroke rate led to maximum swimming speed. Strictly, combination of stroke rate and stroke length determines swimming speed (v = SR·SL). For that reason, most swimmers try to increase SR when SL starts to decrease due to fatigue (Alberty et al., 2009; Craig et al., 1985; Keskinen and Komi, 1993; Pelayo et al., 2007). If they do not achieve it, their swimming speed decreases (Alberty et al., 2005). In the present study, rest between consecutive trials was five minutes, so fatigue did not appear. As expected, v and SL dropped when load increased, due to the increased drag. Significant drop compared to free swimming was observed in these variables from the first load. On the other hand, SR did not change significantly when speed (and load) did. This was consistent with the studies conducted by Alberty et al. (2005) and Pelayo et al., (1996). Gourgoulis et al. (2010) reported that SR dropped when swimming with loads compared to free swimming, but no difference was found in SR between loads. However, in some other studies (Alberty et al., 2009; Craig et al., 1985; Keskinen and Komi, 1993; Pelayo et al., 2007) swimmers managed to increase SR when speed started to decrease. This difference is presumably owing to the fact that the limiting factor in our case was not fatigue, but load. There was not a point where v, SL or SR trends clearly changed (Fig. 1), but it is interesting to observe that they all intersected close to 1m/s, around 2.84kg load.

To the best of our knowledge, there are no studies which have compared intra-cycle speed while semi-tethered swimming, pulling different loads. We observed three main patterns (Fig. 2). Only for the first loads, up to 4.71kg, swimmers were able to keep a constant and relatively high average speed (0.9m/s) after a sharp decrease due to the impulse from the wall. In the rest of trials, excessive load made average 3 strokes speed drop to 0.5–0m/s. Speed reduction was linear and longer in time until swimmers reached a stable speed for next two loads. In the last trials, load was too high for the swimmers to keep any constant speed, so it decreased gradually during the whole trial until 0m/s.

To the authors’ knowledge, no previous investigation has analysed speed during propulsive phases while semi-tethered swimming. Shionoya et al. (1999) assessed average speed during semi-tethered swimming with several loads: 1, 4, 7 and 10kg. The values obtained were: 1.34, 1.07, 0.79 and 0.45m/s, which are similar to our propv_peak_ data, considering that loads were slightly different. In the present study, peak speed was significantly higher than mean speed during propulsive phases in semi-tethered swimming (p<0.05). Like in stroke parameters, significant decrease compared to zero load was observed in propv_mean_ and propv_peak_ from the first resisted condition. In contrast, no significant change in peak propulsive speed was observed over 5.96kg, but this was not enough to enable swimmers to reach a stable speed during a trial. This stagnation of propv_peak_ may be owing to the fact that, despite having their legs tied, most swimmers tried to move them for stabilization when swimming with the highest loads, what turned into a bigger propulsion and higher speed. Despite this, there was a high correlation between load and peak speed (r = −0.971, p<0.01). On the other hand, significant change in %v compared to no load condition was first noticed with 4.71kg. This was also the last load with which swimmers could keep a constant speed during the whole trial. As a whole, the higher the load, the lower the mean and peak speed of propulsive phases and the bigger the relative difference between them (%v). This means that intra-cycle speed variations became larger with higher loads. This may have happened because the swimmers may have tried to jerk to move forward pulling too heavy loads.

Skilled swimmers increased IdC when speed increased while swimming free (Schnitzler, et al., 2010; Schnitzler, et al., 2008) or when speed decreased while swimming with added resistance (parachute, paddles or both) (Schnitzler et al., 2011; Telles et al., 2011). In agreement with this, in the present study IdC increased with growing load and decreasing velocity. Significant change compared to free swimming first happened with 2.84kg. This change in coordination is probably the consequence of the swimmers’ adaptations to higher drag minimizing energy costs. They enhanced relative duration of propulsive phases (pull+push) (Gourgoulis et al., 2010) and overlapped propulsive forces of both arms to overcome increased drag (Maglischo et al., 1984). Semi-resisted training may be, therefore, useful to change coordination mode to superposition or to consolidate it, which has been proved to be the more widely used by expert swimmers (Seifert et al., 2004).

Resisted training in swimming enhanced swimming speed (Girold et al., 2006; Mavridis et al., 2006) and strength (Girold et al., 2006; Girold et al., 2007). Conversely, after comparing tethered and non-tethered stroke mechanics, it was concluded that repeated tethered training would entail detrimental adjustments in swimming technique and, therefore, swimmers’ performance would probably deteriorate (Maglischo et al., 1984). Nevertheless, no negative changes would be expected if tethered swimming was only a part of the training program (Maglischo et al., 1985). According to Shionoya et al. (1999), the most suitable load for training is the load which produces the maximum power in the force-power curve. Further research is required to determine whether a relationship between swim power production and stroke and coordination parameters exists.

Summing up, the most interesting findings of this study were that, over 4.71kg load, a constant swimming speed could not be maintained during a short period of time, and differences between mean and peak propulsive speed were significantly higher than in free swimming. Besides, IdC was found to increase with loads, significantly over 2.84kg. In light of the results, it is suggested that optimal load for resisted training in swimming should be individually determined between 2.84 and 4.71kg (swimming speed between 0.91 and 0.54m/s, respectively).

As a concluding remark, it can be stated that semi-tethered swimming is one training method to enhance swimmers’ performance, although load needs to be carefully controlled. Our results showed that stroke and coordination parameters were not modified to a great extent under certain load. Moreover, resisted training would be beneficial to coordination mode. Training load should be, however, individually determined.

## Figures and Tables

**Table 1:** Pearson′s correlation coefficients between load and the rest of variables.^*^: p<0.01;^ns^: not significant. propv_mean_: mean speed of propulsive stroke phases (pull+push); propv_peak_: peak speed of propulsive stroke phases; %v: percentage of increase from propv_mean_to propv_peak_

	**v (m/s)**	**SR (Hz)**	**SL (m/cic)**	**propv_mean_ (m/s)**	**propv_peak_ (m/s)**	**%v**	**IdC (%)**
**Load**	−0.985^*^	−0.211^ns^	−0.989^*^	−0.984^*^	−0.971^*^	0.922^*^	0.910^*^
